# Pancreatic SBRT in Italian Clinical Practice: A Multicenter Analysis of 473 Patients Treated in 17 Centers

**DOI:** 10.3390/cancers18213594

**Published:** 2026-11-09

**Authors:** Rita Marina Niespolo, Luca Nicosia, Eliana La Rocca, Angela Romano, Fernando Munoz, Alessia Surgo, Bruno Meduri, Pierfrancesco Franco, Sabrina Montrone, Andrea D’Aviero, Elena Galofaro, Francesca De Felice, Alessandra Arcelli, Giampaolo Montesi, Filippo De Renzi, Francesco Romeo Filippone, Marco Lorenzo Bonù, Marco Lupattelli

**Affiliations:** 1 Radiation Oncology Unit, IRCCS San Gerardo dei Tintori, 20900 Monza, Italy; 2 Department of Advanced Radiotherapy Oncology, IRCCS Sacro Cuore-Don Calabria Hospital, Negrar Oncology Care Center, 37024 Verona, Italy; 3 Radiation Oncology Unit, Verona University Hospital, 37126 Verona, Italy; 4 Department of Diagnostic Imaging and Oncological Radiotherapy, Agostino Gemelli University Hospital Foundation, IRCCS, 00168 Rome, Italy; 5 Radiation Oncology Unit, Parini Hospital, 11100 Aosta, Italy; 6 Department of Medicine and Surgery, LUM University, Casamassima, 70010 Bari, Italy; 7 Proton Therapy Unit, Department of Medical Specialties, New Trentino Hospital Center, 38122 Trento, Italy; 8 Department of Translational Medicine (DIMET), University of Eastern Piedmont, 13100 Novara, Italy; pierfrancesco.franco@uniupo.it; 9 Radiotherapy Unit, Pisa University Hospital, Via Roma 69, 56120 Pisa, Italy; 10 Department of Radiation Oncology, Ospedale SS. Annunziata, 66100 Chieti, Italy; 11 Radiation Oncology, University Hospital of the Marche, 60126 Ancona, Italy; 12 Department of Radiological, Oncological and Pathological Sciences, “Sapienza” University of Rome, 00185 Rome, Italy; 13 Radiation Oncology Unit, IRCCS Azienda Ospedaliero-Universitaria di Bologna, 40138 Bologna, Italy; 14 Radiotherapy Unit, Department of Radiological Sciences, Azienda Sanitaria Territoriale Pesaro-Urbino, 61122 Pesaro, Italy; 15 Radiation Oncology, San Martino Hospital, AULSS1 Veneto, 32100 Belluno, Italy; 16 Radiation Oncology Unit, ASST Papa Giovanni XXIII, 24127 Bergamo, Italy; 17 Department of Radiation Oncology, University of Brescia and Civil Hospitals, Piazzale Spedali Civili 1, 25123 Brescia, Italy; marco.bonu@unibs.it; 18 Radiation Oncology Unit, Santa Maria della Misericordia Hospital, 06132 Perugia, Italy

**Keywords:** pancreatic cancer, stereotactic body radiotherapy, MR-guided radiotherapy, ablative radiotherapy, BED10, local control, toxicity, multicenter study

## Abstract

Background/Objectives: Pancreatic adenocarcinoma remains one of the most lethal malignancies, with limited options for durable local control. Stereotactic body radiotherapy (SBRT) has emerged as a promising modality, particularly with the advent of MR-guided radiotherapy (MRgRT) and the possibility of delivering ablative dose levels. This multicenter study aimed to describe real-world Italian practice, evaluate safety and outcomes, and assess the association between ablative dosing, MRgRT, radiological response, and local control. Methods: A retrospective multicenter analysis was conducted across 17 Italian centers, including 473 patients treated with SBRT between 2021 and 2025. Clinical, dosimetric, toxicity, and outcome data were collected. BED10 was calculated for all treatments. Radiological response was assessed using RECIST 1.1 criteria, and toxicity was graded according to CTCAE v5.0. Survival outcomes were estimated using Kaplan–Meier analysis. Results: Median age was 69.6 years, and 59.4% of patients had ECOG performance status 0–1. The study population consisted predominantly of patients with non-metastatic disease, including resectable, borderline resectable, and unresectable locally advanced pancreatic cancer. Most patients received pre-SBRT chemotherapy (78%). The most common regimen was 45 Gy in 5 fractions. MRgRT was used in 20% of cases and was associated with reduced treatment margins (3–5 mm) and improved duodenal sparing (V33 < 0.5 cc in 91%). Acute grade ≥ 3 toxicity was 3.7%, and Late toxicity was uncommon, with grade ≥ 3 events observed in 3.0% of evaluable patients. Radiological response showed complete response in 5%, partial response in 27.7%, stable disease in 44.3%, and progressive disease in 23%, corresponding to an overall disease control rate of 77%. Patients receiving BED10 ≥ 80 Gy achieved higher partial response rates (38% vs. 19%) and lower progression rates (12% vs. 23%) compared with those receiving BED10 < 80 Gy. Median overall survival was 18.4 months, and median progression-free survival (PFS) was 10.2 months. Conclusions: SBRT demonstrated a favorable safety profile and was associated with encouraging clinical outcomes in real-world Italian practice. Ablative dose delivery (BED10 ≥ 80 Gy) was associated with improved radiological response and local control without an apparent increase in toxicity. MRgRT was associated with improved organ-at-risk protection and may facilitate the safe delivery of ablative dose levels. The present findings primarily reflect outcomes in a predominantly non-metastatic population and support the role of SBRT as a component of modern multidisciplinary pancreatic cancer management. This pooled national analysis may serve as the basis for future prospective studies aimed at optimizing patient selection, dose prescription, and image-guided treatment strategies.

## Simple Summary

Pancreatic cancer is one of the most aggressive malignancies, and many patients are not candidates for surgery. In recent years, stereotactic body radiotherapy (SBRT) has become an important treatment option because it enables the delivery of high radiation doses with great precision over a short treatment course. In this multicenter study, we analyzed real-world data from 473 patients treated across seventeen Italian centers, representing the largest Italian experience reported to date. SBRT demonstrated an excellent safety profile, with very low rates of severe toxicity, and was associated with high rates of disease control and local control in a predominantly non-metastatic population. Higher radiation doses (BED10 ≥ 80 Gy) were associated with improved radiological response and local control without an increase in toxicity. Magnetic resonance-guided radiotherapy was associated with improved organ-at-risk protection and may facilitate the safe delivery of ablative dose levels, although both MR-guided and conventional SBRT proved effective in clinical practice. Overall, these findings support the role of SBRT as a safe and effective component of modern multidisciplinary pancreatic cancer management and further support the investigation of ablative treatment strategies in appropriately selected patients.

## 1. Introduction

Pancreatic cancer remains one of the most lethal malignancies worldwide, despite substantial advances in systemic therapy and supportive care. Most patients present with locally advanced or metastatic disease, and only a minority are eligible for potentially curative surgery. In this context, radiotherapy has progressively gained relevance as a local treatment modality, supported by technological developments that enable the safe delivery of high and potentially ablative radiation doses in a limited number of fractions. Current international guidelines, including NCCN and ESMO recommendations, recognize the potential role of radiotherapy and treatment intensification within multidisciplinary pancreatic cancer management, particularly in selected patients with locally advanced or borderline resectable disease [1,2].

Stereotactic body radiotherapy (SBRT) has emerged as an attractive treatment option for pancreatic cancer, offering shorter treatment courses, improved integration with systemic therapy, and a generally favorable toxicity profile while achieving encouraging rates of local control. Several prospective and retrospective studies have demonstrated the feasibility and safety of pancreatic SBRT, reporting favorable local control rates and acceptable gastrointestinal toxicity compared with conventional chemoradiotherapy approaches [3,4,5,6,7,8,9,10]. Early studies, including those by Herman et al. and subsequent dose-escalation experiences, suggested that ablative dose levels (biologically effective dose, BED10 ≥ 80–100 Gy) were associated with improved tumor response and local disease control, although careful management of organs at risk, particularly the duodenum and stomach, remains essential [4,6,11,12]. Increasing evidence further suggests a potential association between biologically effective dose escalation and improved local control and survival outcomes in pancreatic cancer [11,12].

The introduction of magnetic resonance-guided radiotherapy (MRgRT) has further expanded the possibilities of pancreatic SBRT. MR-guided platforms allow direct tumor visualization, reduced target margins, respiratory motion monitoring, and online adaptive planning. Previous studies have suggested that MR-guided radiotherapy may facilitate safer delivery of ablative dose levels through improved visualization and daily adaptation, potentially reducing gastrointestinal toxicity while maintaining adequate target coverage [13,14,15,16,17]. Moreover, the development of online adaptive workflows has enabled rapid treatment re-optimization immediately before irradiation, improving compliance with organ-at-risk constraints while preserving target coverage [13,18]. More recently, prospective studies have confirmed the feasibility and promising clinical outcomes of stereotactic MR-guided adaptive radiotherapy (SMART) in both locally advanced and borderline resectable pancreatic cancer [19].

Despite these advances, evidence remains limited by relatively small patient cohorts, heterogeneous treatment protocols, and modest representation of routine clinical practice. Large multicenter analyses reflecting contemporary real-world management and encompassing different technologies, dose schedules, toxicity profiles, radiological responses, and clinical outcomes remain scarce. Recent systematic reviews have confirmed the growing evidence supporting pancreatic SBRT while highlighting the need for larger real-world datasets and greater standardization of treatment approaches [20,21].

To address this gap, we conducted the largest Italian multicenter analysis of pancreatic SBRT, including 473 patients treated across 17 centers using conventional and MR-guided techniques. The study population included patients treated across different clinical scenarios, including resectable, borderline resectable, unresectable locally advanced, recurrent, and selected oligometastatic disease.

The primary aim of this study was to provide a comprehensive overview of contemporary Italian clinical practice, including treatment patterns, technological approaches, toxicity, radiological response, local control, and survival outcomes. In addition, we explored the association of ablative dose delivery (BED10 ≥ 80 Gy) and MR-guided radiotherapy with treatment response and clinical outcomes.

This analysis provides an updated benchmark of national practice and may support future prospective studies aimed at optimizing patient selection, dose prescription, and image-guided treatment strategies in pancreatic SBRT.

## 2. Materials and Methods

### 2.1. Study Design and Setting

A retrospective multicenter analysis was conducted across 17 Italian radiation oncology centers and included patients with pancreatic adenocarcinoma treated with stereotactic body radiotherapy (SBRT) between January 2021 and December 2025. The primary objective was to characterize real-world Italian practice, describing patient management, technological approaches, treatment patterns, toxicity, radiological response, and oncological outcomes.

### 2.2. Patient Selection

Eligible patients met the following inclusion criteria:

- histologically confirmed pancreatic adenocarcinoma; treatment with stereotactic body radiotherapy (SBRT) delivered with definitive, neoadjuvant, or consolidative intent; treatment intent was recorded by participating centers according to multidisciplinary tumor board recommendations.
- In patients with stage IV disease, SBRT was primarily delivered for local control of the primary pancreatic lesion within the context of oligometastatic, oligoprogressive, or otherwise controlled metastatic disease whenever considered clinically appropriate.
- availability of baseline imaging and complete dosimetric data;
- minimum follow-up of 3 months, unless death occurred earlier.

Consecutive patients meeting the eligibility criteria were included. Exclusion criteria included non-adenocarcinoma histologies, incomplete clinical or dosimetric datasets, and stereotactic radiotherapy delivered for extra-pancreatic oligometastatic lesions. After application of the inclusion and exclusion criteria, 473 patients were available for the final analysis. Disease status at the time of SBRT was categorized as resectable, borderline resectable, unresectable locally advanced disease, oligometastatic stage IV disease, or local recurrence according to data recorded by each participating institution. SBRT indications and treatment intent were determined through multidisciplinary evaluation and reflected real-world clinical practice across participating centers.

### 2.3. Clinical and Tumor Characteristics

Clinical, pathological, and treatment-related data were retrospectively collected from institutional databases and medical records. Collected variables included age, sex, ECOG performance status, disease status at the time of SBRT (resectable, borderline resectable, unresectable locally advanced disease, oligometastatic stage IV disease, or local recurrence), primary tumor location (head, body, or tail of the pancreas), serum CA 19-9 levels before and after SBRT, comorbidities, previous pancreatic surgery, baseline symptoms, clinical response, and details of systemic therapy administered before SBRT, including treatment regimen, number of cycles delivered before radiotherapy, and response to induction chemotherapy.

Data regarding systemic therapies administered after SBRT were not systematically collected across participating institutions and were therefore not included in the present analysis. Systemic treatment before SBRT was delivered according to institutional practice and multidisciplinary tumor board recommendations. The most commonly administered regimens included FOLFIRINOX and gemcitabine plus nab-paclitaxel. When available, information regarding subsequent surgical resection, patterns of disease progression, and follow-up status was also recorded. For patients presenting with stage IV disease, additional information regarding metastatic burden, metastatic disease status at the time of SBRT (oligometastatic, oligoprogressive, or polymetastatic), treatment indication, and therapeutic intent was collected whenever available.

### 2.4. Simulation and Treatment Planning

SBRT was delivered using conventional image-guided linear accelerators or MR-guided radiotherapy systems. Simulation procedures included four-dimensional computed tomography (4D-CT) and/or magnetic resonance imaging (MRI), according to institutional practice. Respiratory motion management strategies included active breathing control, respiratory monitoring, abdominal compression, or other motion mitigation techniques available at each participating center. Target volume definition and treatment planning were performed according to institutional protocols and contemporary stereotactic radiotherapy recommendations. Treatment plans were optimized to achieve adequate target coverage while respecting dose constraints for organs at risk (OARs), with particular attention to gastrointestinal structures and duodenal exposure. Dose constraints and planning objectives were based on institutional practice and published stereotactic radiotherapy guidelines, including AAPM Task Group 101 recommendations and the UK consensus on normal tissue dose constraints for stereotactic radiotherapy [22,23]. When available in institutional records, duodenal sparing was assessed using a V33 constraint of <0.5 cc.

Given the multicenter nature of the study, simulation procedures, motion-management strategies, and planning approaches reflected routine clinical practice at participating institutions while adhering to contemporary SBRT recommendations.

### 2.5. Dose and Fractionation

Prescribed doses ranged from 24 to 67.5 Gy. The vast majority of patients were treated with 5-fraction SBRT schedules, whereas only a small subset received treatment in 3 fractions (24–26 Gy). The most commonly prescribed regimen was 45 Gy delivered in 5 fractions, followed by 30 Gy in 5 fractions and 50 Gy in 5 fractions. The 45 Gy/5-fraction regimen corresponds to a BED10 of 85.5 Gy, exceeding the predefined ablative threshold of BED10 ≥ 80 Gy.

Dose prescription and fractionation were individualized according to target characteristics, proximity to organs at risk, and institutional practice.

The biologically effective dose (BED10) was calculated using the linear–quadratic model according to the following formula: BED10 = nd [1 + d/(α/β)], where n is the number of fractions, d is the dose per fraction, and α/β was assumed to be 10 Gy for pancreatic cancer. BED10 values were categorized as BED10 < 80 Gy or BED10 ≥ 80 Gy (ablative dose).

The threshold of BED10 ≥ 80 Gy was selected to identify ablative treatment schedules, consistent with previous studies suggesting an association between dose escalation and improved local control and survival outcomes in pancreatic cancer [11,12].

BED10 values were recorded for both the gross tumor volume (GTV) and planning target volume (PTV) and were subsequently analyzed according to their association with oncological outcomes. For subgroup analyses, patients were categorized according to the BED10 prescribed to the PTV (BED10 ≥ 80 Gy versus BED10 < 80 Gy).

### 2.6. Image Guidance and Adaptive Radiotherapy

Daily image guidance consisted of cone-beam computed tomography (CBCT) for conventional SBRT treatments and real-time cine magnetic resonance imaging (MRI) for MR-guided radiotherapy. Online adaptive replanning was available for MR-guided treatments and was performed when organ-at-risk (OAR) constraints were exceeded or target coverage was considered suboptimal. Treatment adaptation aimed to maintain adequate target coverage while respecting predefined dose constraints for adjacent critical structures. The adaptive workflow adopted in participating centers reflects approaches previously described for stereotactic MR-guided adaptive radiotherapy in pancreatic cancer [13,18]. Given the multicenter nature of the study, image-guidance strategies and adaptive radiotherapy workflows reflected routine clinical practice at participating institutions, while adhering to contemporary recommendations for pancreatic SBRT.

### 2.7. Toxicity Assessment

Treatment-related toxicity was assessed according to the Common Terminology Criteria for Adverse Events (CTCAE), version 5.0. Toxicity evaluation and reporting were performed in accordance with contemporary recommendations for pancreatic stereotactic body radiotherapy and established SBRT quality assurance guidelines [20,22,24]. Acute toxicity was defined as any treatment-related adverse event occurring within 6 months after completion of SBRT, whereas late toxicity was defined as events occurring thereafter. Toxicity was graded according to CTCAE v5.0 and categorized as gastrointestinal (including nausea, vomiting, diarrhea, abdominal pain, ulceration, bleeding, or perforation), biliary, pain-related, or systemic adverse events. All available toxicity data were retrospectively collected from institutional medical records and follow-up assessments.

Given the retrospective multicenter design, toxicity reporting reflected routine clinical practice at participating institutions and was subsequently harmonized using CTCAE v5.0 criteria to ensure consistent toxicity classification across centers.

### 2.8. Radiological and Biochemical Response

Radiological response was evaluated using contrast-enhanced computed tomography (CT), magnetic resonance imaging (MRI), and/or positron emission tomography (PET), according to institutional follow-up protocols. No uniform predefined post-treatment imaging time point was mandated across participating centers, and radiological response assessment reflected routine institutional follow-up practice. Given the known challenges associated with post-SBRT evaluation in pancreatic cancer, radiological findings were interpreted within the clinical context and with awareness of treatment-related fibrosis, inflammatory changes, and imaging-related variability.

Tumor response was assessed using the Response Evaluation Criteria in Solid Tumors (RECIST), version 1.1 [25], and classified as complete response (CR), partial response (PR), stable disease (SD), or progressive disease (PD).

Given the multicenter nature of the study, imaging modalities and follow-up schedules were selected according to institutional practice. RECIST v1.1 criteria were applied locally at each participating center, and no central radiological review was performed. When more than one imaging modality was available, response assessment was based on the evaluation reported by the treating institution. The first available post-treatment imaging evaluation was considered the initial response assessment.

### 2.9. Study Endpoints

The primary endpoint of the study was overall survival (OS). Secondary endpoints included:

- progression-free survival (PFS), defined as the interval between completion of SBRT and documented local progression, distant progression, combined local and distant progression, or death from any cause, whichever occurred first. Patients without disease progression were censored at the date of last follow-up. Given the inclusion of patients with unresectable, recurrent, and metastatic disease, PFS was considered a more appropriate endpoint than disease-free survival (DFS).
- local failure was defined as radiological evidence of progression within the treated pancreatic target volume according to available follow-up imaging and multidisciplinary assessment.
- local control (LC) was defined as the absence of local failure within the irradiated pancreatic lesion.
- radiological response according to RECIST version 1.1 criteria.
- acute and late treatment-related toxicity.
- the association of ablative dose delivery (BED10 ≥ 80 Gy) with treatment outcomes.
- comparison of clinical outcomes between MR-guided and conventional SBRT.

Survival outcomes, local control, radiological response, and toxicity were evaluated in the overall study population and according to BED10 category and radiotherapy technique.

Survival analyses were performed using PFS and OS as the principal oncological outcome measures.

The selected endpoints were chosen to reflect the principal efficacy and safety outcomes commonly reported in contemporary pancreatic SBRT studies, prospective clinical series, and systematic reviews [4,20,21].

### 2.10. Statistical Analysis

Descriptive statistics were used to summarize patient, tumor, and treatment characteristics. Continuous variables were reported as median (range), whereas categorical variables were expressed as frequencies and percentages.

Overall survival (OS), progression-free survival (PFS), and local control (LC) were evaluated as the main oncological outcomes. OS was calculated from the date of SBRT completion to death from any cause or last follow-up. Patients alive at the time of last follow-up were censored. PFS was calculated from the date of SBRT completion to disease progression or death from any cause, whichever occurred first. LC was calculated from the date of SBRT completion to radiologically documented local progression within the irradiated volume. Patients without local progression were censored at the time of their last available radiological assessment.

OS, PFS, and LC were estimated using the Kaplan–Meier method. Comparisons between groups (MR-guided versus conventional SBRT and BED10 ≥ 80 Gy versus BED10 < 80 Gy) were performed using the log-rank test.

Categorical variables were compared using the χ^2^ test or Fisher’s exact test, as appropriate, whereas continuous variables were analyzed using the Mann–Whitney U test.

Missing data were not imputed and were handled using available-case analysis. Denominators were reported according to variable-specific data availability whenever applicable.

All statistical tests were two-sided, and a *p*-value < 0.05 was considered statistically significant. Given the retrospective and exploratory nature of the study, all analyses should be considered hypothesis-generating.

Statistical analyses were performed using IBM SPSS Statistics version 29.0 (IBM Corp., Armonk, NY, USA).

## 3. Results

### 3.1. Baseline Characteristics

A total of 473 patients treated with pancreatic SBRT across 17 Italian centers were included in the analysis. The median age was 69.6 years (range, 33–90 years), and the sex distribution was balanced (48.0% male and 52.0% female). Most patients had an ECOG performance status of 0–1 (59.4%).

Disease status at the time of SBRT was classified as resectable in 72 patients (15.2%), borderline resectable in 91 patients (19.2%), unresectable locally advanced disease in 238 patients (50.3%), oligometastatic stage IV disease in 31 patients (6.6%), and local recurrence in 30 patients (6.3%). The study population therefore consisted predominantly of patients with non-metastatic disease, while a limited proportion of patients presented with oligometastatic stage IV disease. This distribution reflects the real-world multicenter nature of the cohort, including patients treated with SBRT across diverse clinical scenarios and therapeutic intents, including neoadjuvant, definitive, and consolidative approaches.

Tumors were predominantly located in the pancreatic head (62%). Pre-SBRT systemic therapy was administered in 78% of patients, most commonly FOLFIRINOX or gemcitabine plus nab-paclitaxel, according to multidisciplinary evaluation and institutional treatment protocols.

Among patients with initially resectable, borderline resectable, or locally advanced disease, 41 patients (10.2%) subsequently underwent surgical resection after SBRT, reflecting the role of SBRT within multimodality treatment strategies.

Post-SBRT systemic treatment data were not systematically available and were therefore not analyzed.

Baseline patient characteristics are summarized in Table 1, whereas treatment characteristics and clinical outcomes are reported in Table 2 and Table 3.

Disease status at the time of SBRT was categorized as resectable, borderline resectable, unresectable locally advanced disease, oligometastatic stage IV disease, or local recurrence according to clinical presentation.

PFS was defined as the interval from SBRT completion to documented local progression, distant progression, combined local and distant progression, or death from any cause, whichever occurred first.

The study population consisted predominantly of patients with non-metastatic disease, including resectable, borderline resectable, and unresectable locally advanced pancreatic cancer, while only 31 patients (6.6%) had oligometastatic stage IV disease.

### 3.2. Treatment Characteristics

The prescribed dose ranged from 24 to 67.5 Gy delivered in 3–6 fractions. The most frequently adopted regimen was 45 Gy in 5 fractions, corresponding to a BED10 of approximately 85.5 Gy. Dose prescriptions covered a broad spectrum of BED10 values and were subsequently categorized according to the predefined threshold of 80 Gy to distinguish non-ablative and ablative treatments. Conventional image-guided SBRT represented the predominant treatment modality, accounting for approximately 80% of cases, whereas MR-guided radiotherapy was used in about 20% of patients. Planning target volume margins ranged from 3 to 5 mm in MR-guided treatments and from 5 to 7 mm in conventional SBRT. Daily image guidance was performed using cone-beam CT for conventional treatments and real-time cine MRI for MR-guided treatments, with online adaptive replanning available exclusively on MR-guided platforms. MR-guided treatments incorporated reduced margins and online adaptive replanning, with high rates of compliance with gastrointestinal dose constraints observed in this subgroup.

Given the multicenter nature of the study, dose prescriptions, fractionation schedules, and treatment-planning strategies reflected routine clinical practice across participating institutions while remaining consistent with contemporary pancreatic SBRT recommendations.

Treatment characteristics are summarized in Table 2.

### 3.3. Toxicity

Acute toxicity was predominantly mild, with grade ≥ 3 events observed in 17 of 455 evaluable patients (3.7%). The most frequently reported acute adverse events were abdominal pain, nausea/vomiting, and diarrhea, mainly of grade 1–2 severity.

Late toxicity was infrequent and largely limited to grade 1–2 gastrointestinal or biliary events. Grade ≥ 3 late toxicity was observed in 12 of 405 evaluable patients (3.0%).

**Figure 1:**
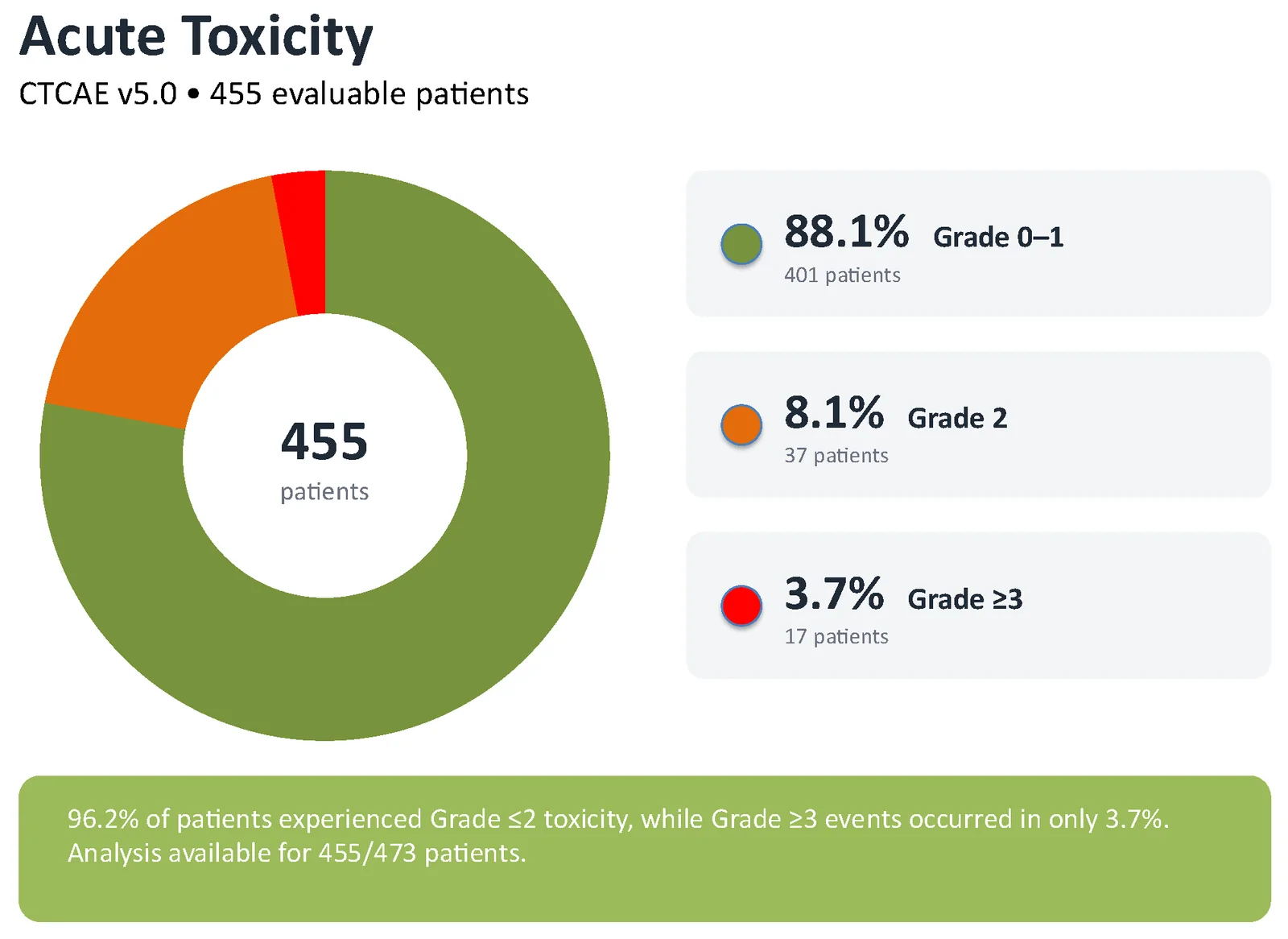
Distribution of Acute Toxicity According to CTCAE v5.0 Following Pancreatic SBRT.

**Figure 2:**
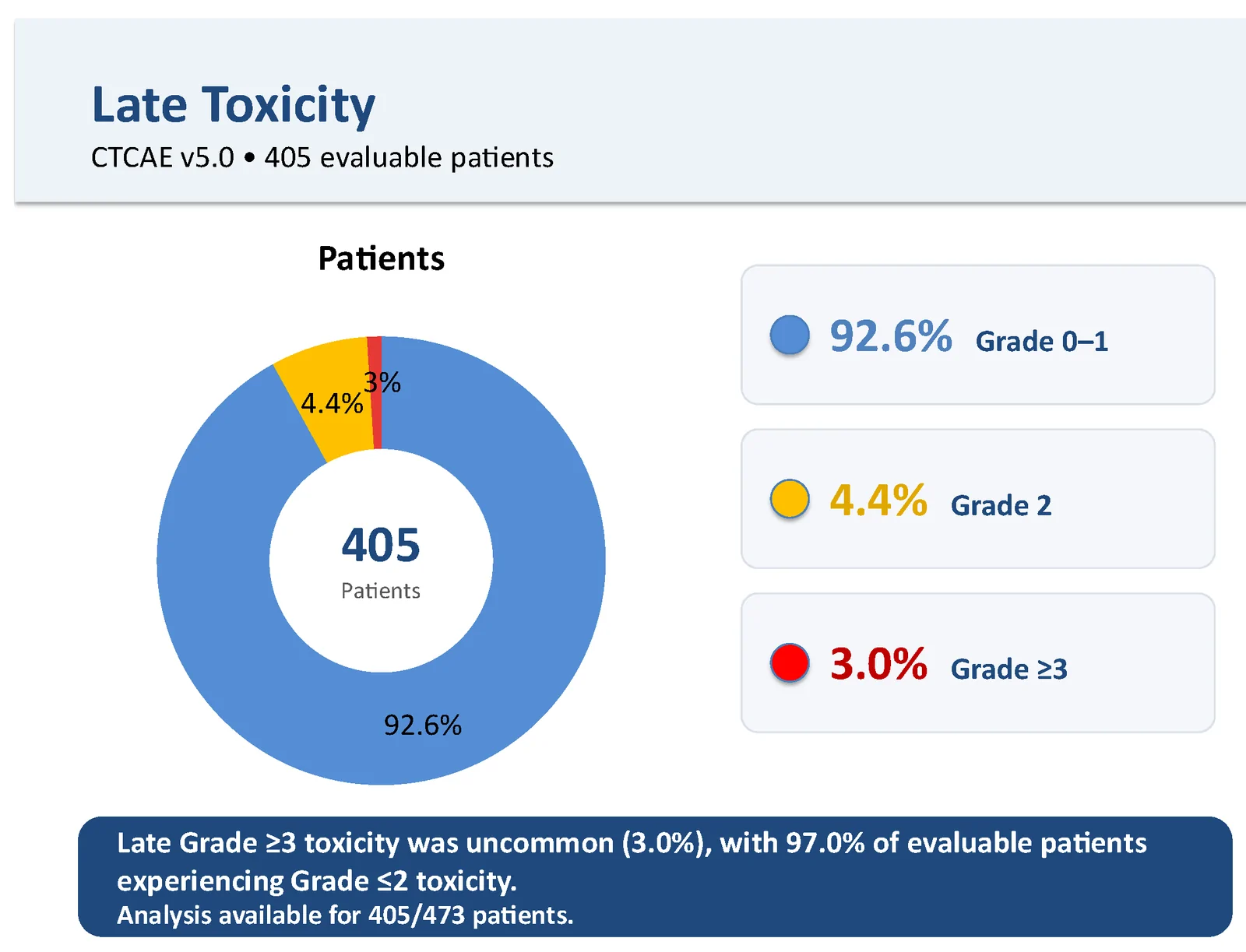
Distribution of Late Toxicity According to CTCAE v5.0 Following Pancreatic SBRT.

No treatment-related duodenal perforation or gastrointestinal bleeding was observed during follow-up. Detailed toxicity distributions are presented in Figure 1 and Figure 2.

Despite the multicenter nature of the study, severe treatment-related toxicity remained uncommon across participating institutions, consistent with the favorable safety profile reported for contemporary pancreatic SBRT in routine clinical practice.

Distribution of acute toxicity (≤6 months) according to CTCAE v5.0 among 473 patients treated with pancreatic stereotactic body radiotherapy (SBRT). Among the 455 evaluable patients, grade 0–1 toxicity was observed in 401 patients (88.1%), grade 2 toxicity in 37 patients (8.1%), and grade ≥ 3 toxicity in 17 patients (3.7%). Eighteen patients were not evaluable for acute toxicity assessment.

Late toxicity was limited and predominantly low grade, with grade 0–1 events reported in 375 of 405 evaluable patients (92.6%). Grade 2 toxicity occurred in 18 patients (4.4%), while grade ≥ 3 late adverse events were observed in 12 patients (3.0%). No major gastrointestinal complications, including ulceration, perforation, or duodenal bleeding, were observed during follow-up. The distribution of late toxicity is illustrated in Figure 2.

Distribution of late toxicity (>6 months) according to CTCAE v5.0 among 473 patients treated with pancreatic stereotactic body radiotherapy (SBRT). Among the 405 evaluable patients, grade 0–1 toxicity was reported in 375 patients (92.6%), grade 2 toxicity in 18 patients (4.4%), and grade ≥ 3 toxicity in 12 patients (3.0%). No major gastrointestinal complications, including ulceration, perforation, or bleeding, were observed during follow-up. Sixty-eight patients were not evaluable for late-toxicity assessment.

### 3.4. Radiological and Biochemical Response

Post-treatment radiological assessment according to RECIST version 1.1 [25] was available for 422 evaluable patients. Complete response was observed in 21/422 patients (5.0%), partial response in 117/422 patients (27.7%), stable disease in 187/422 patients (44.3%), and progressive disease in 97/422 patients (23.0%), resulting in an overall disease control rate (CR + PR + SD) of 325/422 patients (77.0%).

Biochemical response data were not available for the entire study population and were therefore analyzed only in patients with available follow-up CA 19-9 assessments, according to institutional practice.

**Figure 3:**
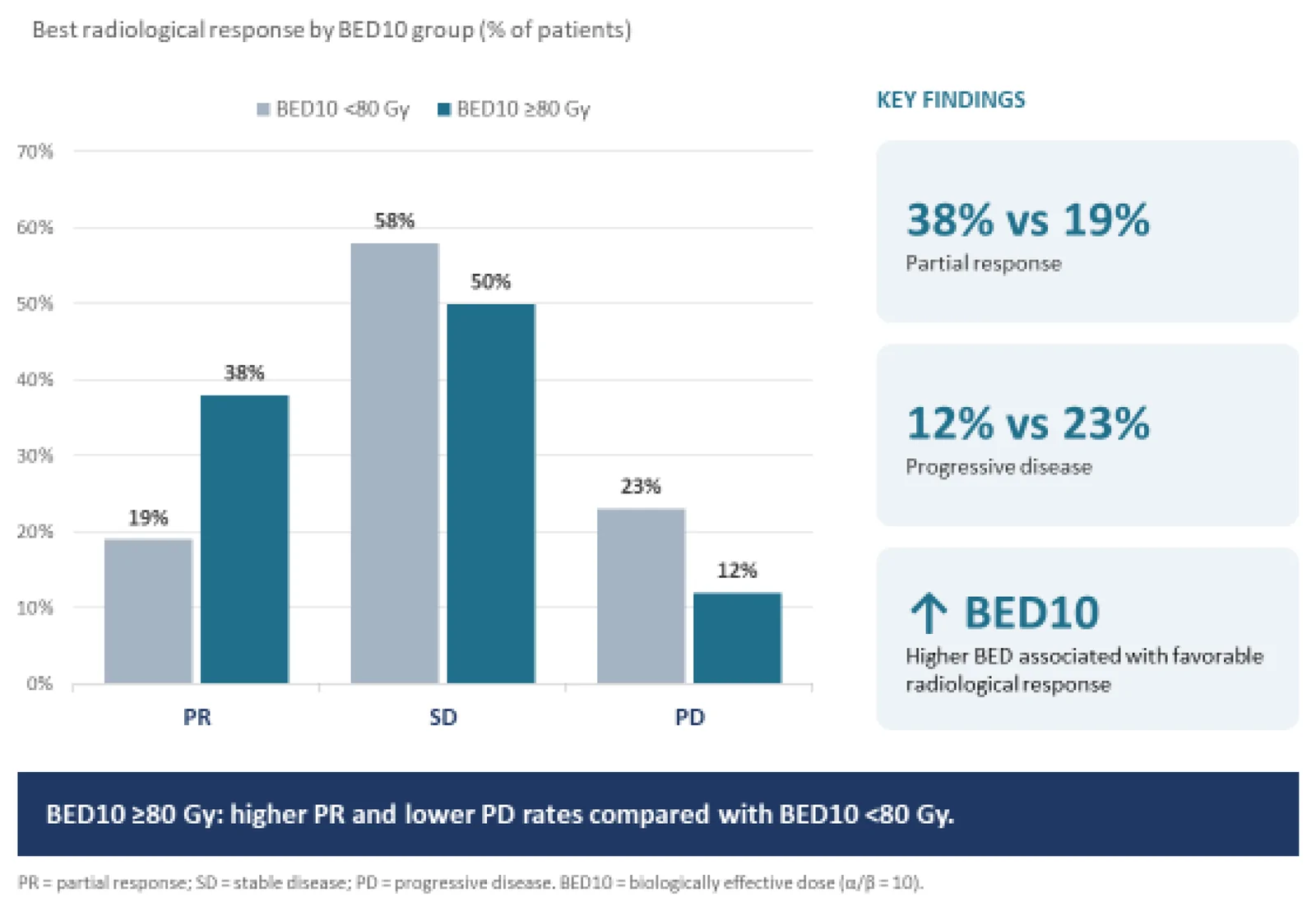
RECIST Response According to BED10 Category.

**Figure 4:**
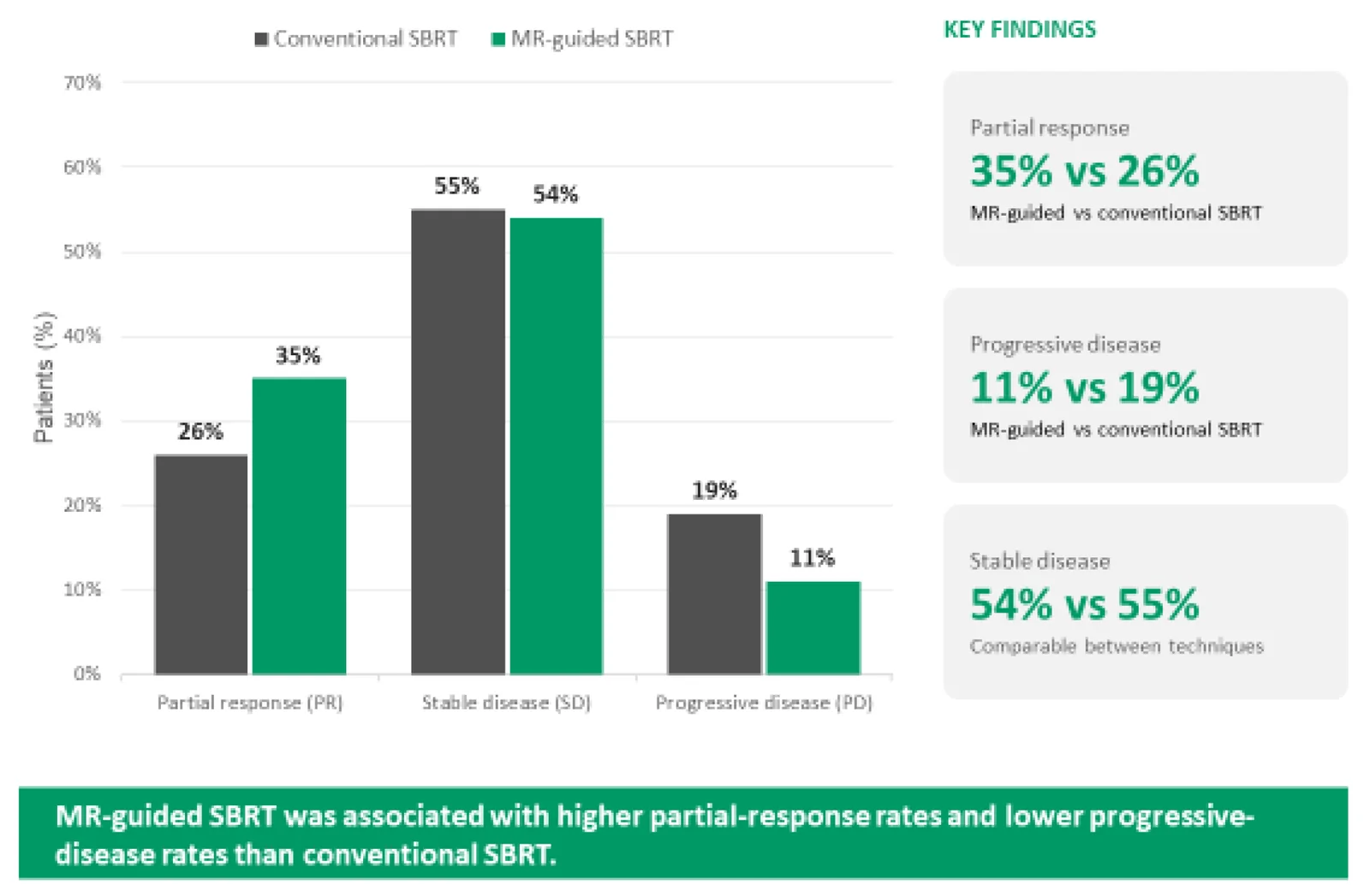
Radiological Response by Radiotherapy Technique.

Radiological response outcomes are illustrated in Figure 3 and Figure 4.

Despite variations in follow-up imaging modalities and schedules across participating institutions, response assessment was standardized according to RECIST version 1.1 criteria, ensuring consistency in radiological outcome evaluation across the multicenter cohort.

Radiological response according to RECIST 1.1 in patients treated with BED10 < 80 Gy and BED10 ≥ 80 Gy. Patients receiving ablative-dose SBRT (BED10 ≥ 80 Gy) showed higher partial response rates (38% vs. 19%) and lower rates of progressive disease (12% vs. 23%) compared with those receiving BED10 < 80 Gy.

Radiological response according to RECIST version 1.1 in patients treated with MR-guided and conventional stereotactic body radiotherapy. MR-guided radiotherapy was associated with a higher proportion of partial responses (35% vs. 26%) and a lower proportion of progressive disease (11% vs. 19%) compared with conventional SBRT.

### 3.5. Clinical Outcomes

After a median follow-up of 13.2 months, median overall survival (OS) was 18.4 months, with 12-month and 24-month OS rates of 62% and 28%, respectively. Median progression-free survival (PFS) was 10.2 months, with corresponding 12-month and 24-month PFS rates of 41% and 19%, respectively. The 12-month local control rate was 82%. Overall, these findings indicate encouraging disease control and survival outcomes in a predominantly non-metastatic population treated with pancreatic SBRT in routine clinical practice.

### 3.6. Impact of Ablative Dose (BED10 ≥ 80 Gy)

**Figure 5:**
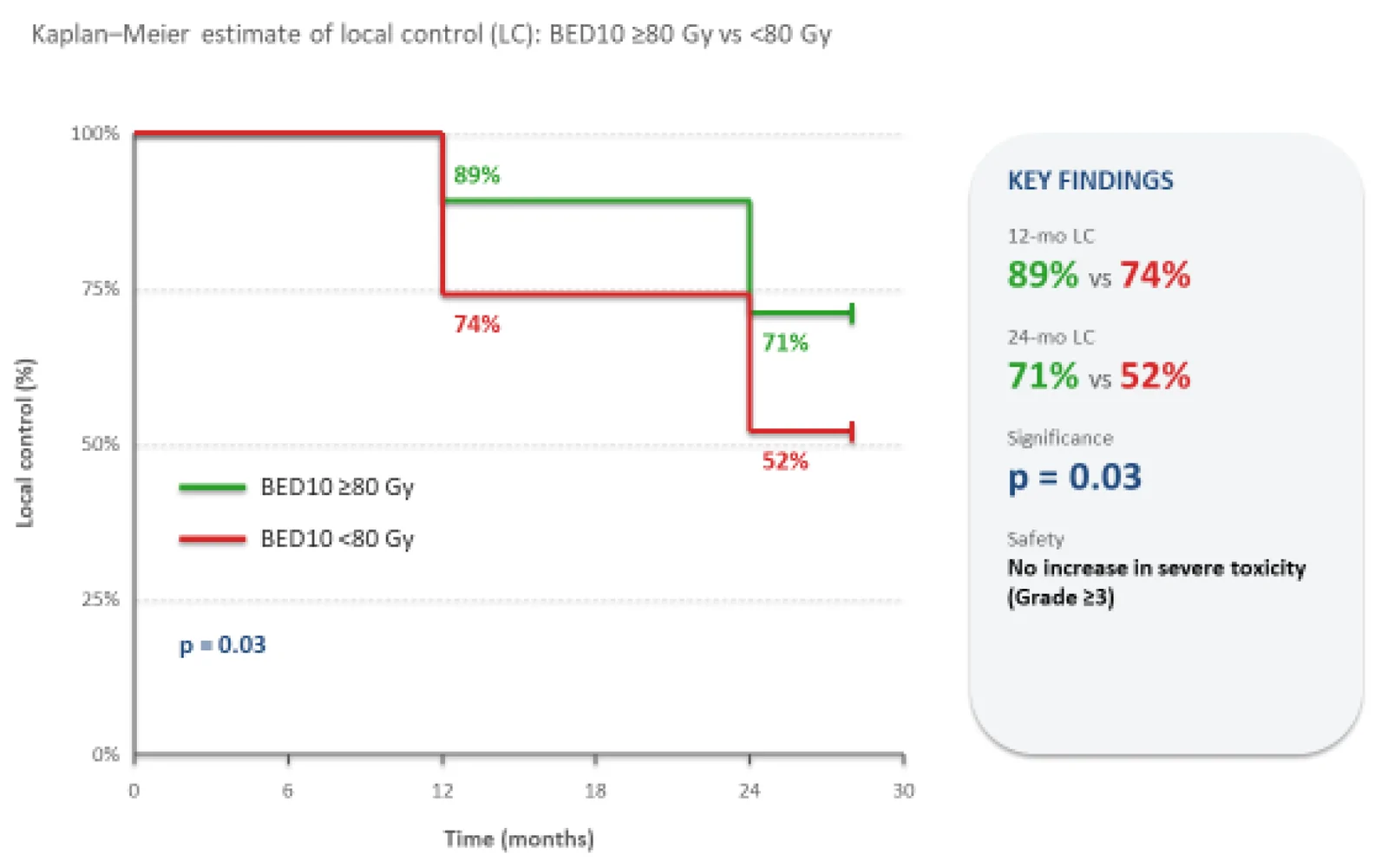
Local Control Stratified by BED10.

Ablative dose delivery (BED10 ≥ 80 Gy) was associated with improved radiological response and local control. Compared with patients receiving BED10 < 80 Gy, those treated with BED10 ≥ 80 Gy showed higher partial response rates (38% vs. 19%) and lower rates of progressive disease (12% vs. 23%), without an apparent increase in severe toxicity. These findings are in line with previous evidence supporting a potential role for dose escalation in improving local tumor control [11,26]. Nevertheless, because of the retrospective design and the potential influence of selection bias and treatment-related confounders, no causal relationship between higher BED10 and improved clinical outcomes can be established. Local control outcomes according to BED10 category are shown in Figure 5.

These findings are consistent with previous evidence suggesting an association between dose escalation, improved local control, and favorable oncological outcomes in pancreatic cancer.

Kaplan–Meier curves of local control according to BED10 category. Ablative dose delivery (BED10 ≥ 80 Gy) was associated with improved local control compared with BED10 < 80 Gy. Local control rates at 12 and 24 months were 89% and 71% for BED10 ≥ 80 Gy versus 74% and 52% for BED10 < 80 Gy, respectively (log-rank *p* = 0.03). No increase in grade ≥ 3 toxicity was observed with ablative dose delivery.

### 3.7. Comparison Between MR-Guided and Conventional SBRT

**Figure 6:**
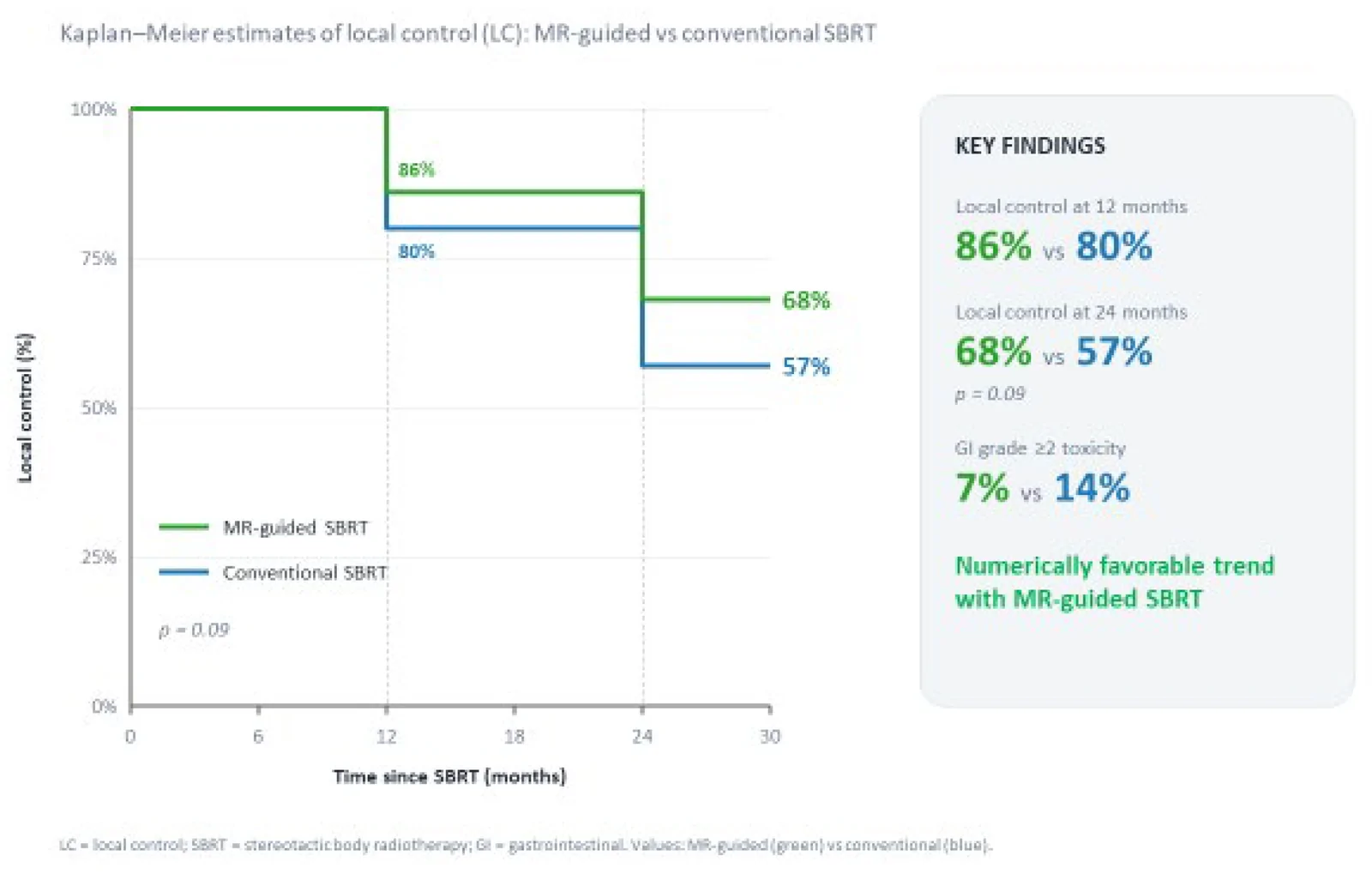
Local Control by Radiotherapy Technique.

MR-guided radiotherapy was characterized by the use of reduced treatment margins, real-time MRI guidance, and the availability of online adaptive replanning [14,15,16,18,19,27]. Compared with conventional SBRT, MR-guided treatments were associated with a trend toward higher partial response rates and lower rates of progressive disease, while maintaining a favorable toxicity profile. No significant differences were observed in overall survival (OS) or progression-free survival (PFS) between treatment techniques. Technique-specific local control outcomes are presented in Figure 6. Although no statistically significant differences in OS or PFS were observed, MR-guided radiotherapy incorporates technical features such as reduced treatment margins, daily soft-tissue visualization, and online adaptive replanning, which may facilitate treatment personalization and organ-at-risk sparing.

Kaplan–Meier estimates of local control stratified by radiotherapy technique. Local control at 12 and 24 months was 86% and 68%, respectively, in the MR-guided radiotherapy group, compared with 80% and 57% in the conventional SBRT group. The observed difference was not statistically significant (log-rank *p* = 0.09).

### 3.8. Summary of Findings

Overall, pancreatic SBRT was associated with high rates of disease control and local control, with limited severe toxicity. Ablative dose delivery (BED10 ≥ 80 Gy) was associated with improved radiological response and local control, while MR-guided radiotherapy was characterized by favorable treatment features and encouraging clinical outcomes. These findings support the role of SBRT as a component of modern multidisciplinary pancreatic cancer management across a range of clinical scenarios, including resectable, borderline resectable, unresectable locally advanced, recurrent, and selected oligometastatic disease. Despite the heterogeneity of the study population, favorable local control and safety outcomes were observed in routine clinical practice.

## 4. Discussion

### 4.1. Overview of Findings

In this large multicenter Italian analysis including 473 patients treated across 17 radiation oncology centers, pancreatic stereotactic body radiotherapy (SBRT) was associated with favorable clinical outcomes and a low incidence of severe toxicity, supporting its role as a widely implemented treatment approach in contemporary clinical practice [20,21,24]. The study provides a comprehensive overview of real-world SBRT delivery across a heterogeneous national network, encompassing different technologies, dose prescriptions, motion-management strategies, and image-guidance platforms.

Overall, treatment was associated with low rates of severe toxicity, high disease control rates, and encouraging local control and survival outcomes. Importantly, delivery of ablative dose levels (BED10 ≥ 80 Gy) was associated with improved radiological response and local control without a corresponding increase in severe toxicity, consistent with previous evidence supporting dose escalation in pancreatic cancer [11,26]. Furthermore, MR-guided radiotherapy was characterized by favorable dosimetric features and encouraging clinical outcomes and may facilitate safe dose escalation through improved target visualization and adaptive treatment delivery, in line with recent experiences of adaptive MR-guided pancreatic SBRT [14,15,16,19].

The scale of the cohort and the participation of 17 centers make this analysis one of the largest contemporary real-world experiences of pancreatic SBRT reported in Europe. Following re-evaluation of the study population, the cohort was found to consist predominantly of patients with non-metastatic disease, including resectable, borderline resectable, and unresectable locally advanced pancreatic cancer, whereas only a limited proportion of patients presented with oligometastatic stage IV disease. Consequently, the reported outcomes primarily reflect the role of SBRT within contemporary multidisciplinary management of non-metastatic pancreatic cancer, while also providing insight into selected recurrent and oligometastatic clinical scenarios encountered in routine practice.

### 4.2. Safety and Tolerability

The very low incidence of acute grade ≥ 3 toxicity (3.7%) and the rarity of severe late adverse events observed in this cohort are consistent with the favorable safety profile of pancreatic SBRT reported in contemporary clinical practice [4,10,20,28]. These findings are consistent with those reported in prospective and retrospective series, including the experiences of Herman et al. and Chuong et al., which established SBRT as a feasible treatment approach with acceptable gastrointestinal toxicity when strict organ-at-risk constraints are respected [4,15].

In the present study, no major gastrointestinal complications, such as duodenal perforation, ulceration, or bleeding, were observed during follow-up, further supporting the favorable safety profile of contemporary SBRT strategies. The low rate of severe toxicity may reflect several factors, including advances in image guidance, improved motion-management techniques, increased attention to gastrointestinal dose constraints, and the progressive adoption of adaptive treatment approaches [22,24,29].

In particular, MR-guided treatments were associated with higher adherence to duodenal dose constraints. This observation may be related to improved visualization of target volumes and adjacent organs at risk, as well as the possibility of online adaptive replanning. However, the retrospective design and lack of formal comparative analyses preclude definitive conclusions regarding differences in toxicity between treatment techniques.

These observations suggest that enhanced target visualization and online adaptive replanning may facilitate treatment personalization and organ-at-risk sparing [14,15,16,18,19,27,30]. However, these findings should be interpreted cautiously given the retrospective nature of the analysis.

Despite the multicenter design and the inclusion of patients across different disease-status categories, treatment-related toxicity remained consistently low across participating institutions, consistent with the favorable safety profile reported for contemporary pancreatic SBRT in routine clinical practice.

### 4.3. Radiological Response and Local Control

The observed radiological response profile, characterized by complete response in 5% of patients, partial response in 27.7% of patients and stable disease in 44.3%, is consistent with the biological behavior of pancreatic adenocarcinoma, in which durable disease stabilization often represents a clinically meaningful therapeutic outcome [4,20,21]. Accordingly, the disease control rate of 77% observed in the present cohort further supports the role of SBRT in achieving local disease containment within a multimodality treatment strategy [4,17,28].

Considering the aggressive natural history of pancreatic cancer, maintenance of local stability may contribute to symptom control, preservation of performance status, and continuation of systemic therapy [1,2,5].

A key finding of this analysis is the association between ablative dose delivery (BED10 ≥ 80 Gy) and improved treatment response.

Patients receiving ablative SBRT showed substantially higher partial response rates and lower rates of radiological progression compared with those treated with lower BED levels. These differences were accompanied by improved local control without a corresponding increase in severe toxicity. Although these findings are consistent with previous reports supporting a potential benefit of dose escalation, the retrospective design of the present study does not allow causal inferences. Patients receiving BED10 ≥ 80 Gy may have differed from those treated with lower BED levels with respect to tumor characteristics, anatomical relationships with adjacent organs at risk, treatment indication, institutional practice, and treatment technology. Therefore, the observed association should be interpreted as hypothesis-generating [11,16,26].

Interestingly, the most frequently prescribed regimen across participating centers was 45 Gy in 5 fractions (BED10 ≈ 85.5 Gy), suggesting that ablative treatment schedules have become widely adopted in contemporary Italian clinical practice. This observation reflects an increasing tendency toward safe dose intensification, facilitated by advances in image guidance, motion management, and treatment planning [15,16,19,27].

Our findings are consistent with previous reports from Rudra, Reyngold, and other investigators demonstrating improved local control and, in selected patient populations, favorable survival outcomes following delivery of ablative radiation doses [11,14,16,26]. The present multicenter experience extends these observations to a large real-world national cohort, supporting the feasibility and growing clinical use of contemporary ablative SBRT strategies in routine practice [20,28].

The association observed between BED10 ≥ 80 Gy and improved radiological response and local control is consistent with previous reports supporting the potential value of ablative-dose radiotherapy in pancreatic cancer. Several biological mechanisms, including enhanced tumor cell death, vascular effects, and modulation of the tumor microenvironment, have been proposed in the literature to explain the potential benefits of dose escalation. However, these mechanisms were not specifically investigated in the present study and therefore should not be inferred from our data. Consequently, the observed association should be considered hypothesis-generating and warrants prospective validation.

Importantly, these observations were derived predominantly from patients with resectable, borderline resectable, and unresectable locally advanced pancreatic cancer. Therefore, the favorable association observed between ablative dose delivery and local tumor control should be interpreted primarily within the context of contemporary multidisciplinary management of non-metastatic disease.

### 4.4. Role of MR-Guided Radiotherapy

Magnetic resonance-guided radiotherapy has become an increasingly adopted approach within the contemporary pancreatic SBRT landscape [14,15,16,19,27,30,31].

In the present cohort, MR-guided treatments were associated with favorable dosimetric characteristics, including a higher rate of compliance with predefined gastrointestinal dose constraints, particularly the duodenal V33 constraint. These findings are biologically plausible and may reflect the inherent advantages of MR-guided platforms, such as superior soft-tissue visualization, continuous intrafraction motion monitoring, reduced target margins, and the availability of online adaptive replanning [14,15,18,19,27]. Collectively, these features may facilitate more precise target coverage while maintaining adherence to gastrointestinal organ-at-risk constraints [18,19,29].

MR-guided radiotherapy was also associated with encouraging trends in radiological response and local control, particularly among patients receiving ablative doses (BED10 ≥ 80 Gy). However, these observations should be interpreted cautiously. The retrospective and non-randomized nature of the study precludes causal inference, and residual confounding related to patient selection, disease characteristics, treatment indication, institutional practice, and technology availability cannot be excluded [15,16,19,26,27]. Importantly, although local control rates appeared numerically higher in the MR-guided cohort, the observed difference did not reach statistical significance and therefore cannot be considered evidence of clinical superiority.

Similarly, no statistically significant differences were observed in overall survival or progression-free survival between MR-guided and conventional SBRT approaches. Consequently, while MR-guided radiotherapy represents a promising technological platform that may facilitate treatment personalization and safe delivery of ablative radiation doses, the present analysis does not demonstrate a significant clinical advantage over conventional SBRT. Future prospective studies are warranted to determine whether the dosimetric and technical benefits of MR-guided adaptive radiotherapy ultimately translate into improved long-term oncological outcomes [14,15,16,19,27,30].

### 4.5. Clinical Outcomes

The survival outcomes observed in this study compare favorably with those reported in contemporary pancreatic SBRT series [4,12,17,20,21,28]. Median overall survival of 18.4 months and median progression-free survival (PFS) of 10.2 months further support the role of SBRT as a component of multimodality treatment strategies for pancreatic adenocarcinoma [4,17,20,28]. Furthermore, the 12-month local control rate of 82% is consistent with previous reports indicating that modern SBRT approaches can provide durable local disease management in a substantial proportion of patients [4,17,26,28].

Although MR-guided radiotherapy demonstrated favorable trends in radiological response and local control, no statistically significant differences in overall survival (OS), progression-free survival (PFS), or local control were observed compared with conventional SBRT. These findings suggest that both treatment platforms can achieve satisfactory oncological outcomes when delivered according to contemporary standards and appropriate organ-at-risk constraints [14,15,19,27].

Importantly, our results are consistent with the hypothesis that treatment outcomes may be more strongly influenced by the ability to safely deliver biologically effective ablative dose levels than by the image-guidance platform itself [11,16,26]. In this context, advanced technologies such as MR-guided radiotherapy may provide their greatest clinical value by facilitating safe dose escalation, improving treatment reproducibility, and enhancing organ-at-risk protection rather than directly affecting survival outcomes [15,18,19,27,30].

### 4.6. Clinical Implications

The findings of the present study support the incorporation of SBRT within multidisciplinary treatment strategies for selected patients with pancreatic cancer in routine clinical practice [1,2,24]. Within the context of contemporary systemic therapy, stereotactic radiotherapy may contribute to durable local disease control while maintaining a favorable safety profile [4,20,21,28].

Our data further support the feasibility of delivering modern SBRT approaches across a broad range of clinical settings, including institutions employing different technologies and treatment workflows. The widespread adoption of contemporary fractionation schedules and advanced image-guidance techniques reflects an increasing degree of standardization in pancreatic SBRT practice and may facilitate the implementation of collaborative treatment protocols and future prospective clinical studies [11,15,16,19,26,27,28].

Importantly, the high degree of consistency observed across 17 Italian centers suggests that pancreatic SBRT can be safely implemented on a national scale, with acceptable variability in target delineation, dose prescription, and treatment delivery. Such standardization may contribute to reducing interinstitutional disparities, improving treatment quality, and facilitating the development of future multicenter research initiatives [22,24,29].

Overall, the present study provides a contemporary real-world benchmark for pancreatic SBRT and supports further prospective investigations aimed at optimizing patient selection, refining dose prescription strategies, and clarifying the role of advanced adaptive radiotherapy technologies in improving clinical outcomes [11,16,19,27]

### 4.7. Study Limitations

This study has several limitations that should be acknowledged. First, its retrospective design may have introduced selection bias and limited the ability to fully account for potential confounding factors across participating institutions.

Second, the multicenter nature of the study resulted in variability in simulation procedures, motion-management strategies, treatment-planning approaches, image-guidance workflows, systemic treatment policies, and follow-up schedules. Although these variations reflect real-world clinical practice and enhance the generalizability of the findings, they may also have contributed to interinstitutional differences in treatment delivery and outcome assessment.

An additional limitation relates to systemic treatment data. While information regarding induction chemotherapy before SBRT, including treatment regimen and number of cycles, was collected, post-SBRT systemic therapies were not systematically recorded. Consequently, the potential influence of subsequent systemic treatment on oncological outcomes could not be evaluated and may represent an important unmeasured confounding factor. Future prospective studies should incorporate standardized collection of treatment-sequencing data.

Radiological response and toxicity assessments were performed according to institutional practice and were not centrally reviewed, potentially introducing variability in outcome reporting. Moreover, interpretation of post-SBRT imaging may be challenging because treatment-related fibrosis and inflammatory changes can mimic residual disease or progression. Quality-of-life measures and patient-reported outcomes were not consistently available and therefore could not be included in the analysis.

Further limitations include the clinical heterogeneity of the study population, which encompassed resectable, borderline resectable, unresectable locally advanced, recurrent, and oligometastatic pancreatic cancer treated with different therapeutic intents, including neoadjuvant, definitive, and consolidative approaches. Consequently, the reported outcomes should be interpreted within the context of a real-world cohort comprising distinct prognostic categories and treatment indications. In addition, the retrospective study design precluded consistent stratified analyses across all disease-status categories and limited direct comparison with contemporary surgical classification systems.

Finally, treatment allocation was not randomized and may have been influenced by patient-related, disease-related, institutional, and technological factors. Therefore, the observed associations between treatment technique, ablative dose delivery, and clinical outcomes should be considered hypothesis-generating and should not be interpreted as evidence of causality.

Nevertheless, the large sample size, inclusion of 473 patients treated across 17 centers, and representation of contemporary clinical practice provide a comprehensive overview of pancreatic SBRT implementation in Italy. These characteristics enhance the external validity of the findings and support their relevance for everyday clinical decision-making.

## 5. Conclusions

This multicenter study, representing the largest Italian experience of pancreatic SBRT reported to date, demonstrates that stereotactic body radiotherapy is associated with favorable safety and clinical outcomes in contemporary practice. Across a heterogeneous national network of 17 centers, pancreatic SBRT was associated with encouraging local control rates and low rates of severe toxicity, supporting its role within modern multidisciplinary treatment strategies.

Ablative dose delivery (BED10 ≥ 80 Gy) was associated with improved radiological response and local control outcomes without a corresponding increase in severe toxicity. However, given the retrospective design and potential residual confounding, these findings should be considered hypothesis-generating and do not establish a causal relationship between dose escalation and clinical outcomes.

MR-guided radiotherapy was associated with favorable dosimetric characteristics and encouraging trends in radiological response and local control. Nevertheless, no significant differences in survival outcomes were observed, and definitive conclusions regarding the comparative effectiveness of different treatment platforms cannot be drawn from the present analysis.

Overall, this collaborative national study provides a robust real-world benchmark for pancreatic SBRT and supports continued investigation of dose-escalated and adaptive radiotherapy strategies. Future prospective studies are warranted to refine patient selection, optimize dose prescription, and further define the clinical impact of advanced image-guided and adaptive radiotherapy technologies.

## Figures and Tables

**Table 1:** Baseline patient, tumor, and disease-status characteristics. (N = 473).

Variable	Value
Number of patients	473
Participating centers	17
Age, median (range), years	69.6 (33–90)
Sex	
Male	227 (48.0%)
Female	246 (52.0%)
ECOG performance status	
0	89 (18.8%)
1	192 (40.6%)
2	148 (31.3%)
3	44 (9.3%)
Disease status at time of SBRT	
Resectable	72 (15.2%)
Borderline resectable	91 (19.2%)
Unresectable locally advanced disease	238 (50.3%)
Oligometastatic stage IV disease	31 (6.6%)
Local recurrence	30 (6.3%)
Missing	11 (2.3%)
Primary tumor location	
Head	293 (62.0%)
Body	132 (28.0%)
Tail	48 (10.0%)
Any comorbidity	194 (41.0%)
Previous pancreatic surgery	57 (12.0%)
Pre-SBRT systemic therapy	369 (78.0%)
Most common systemic regimens	FOLFIRINOX; gemcitabine/nab-paclitaxel
Response to induction chemotherapy	
Partial response	32.0%
Stable disease	48.0%
Progressive disease	20.0%
Baseline CA 19-9 available	430/473 (90.9%)

Response to induction chemotherapy was assessed only among patients receiving systemic therapy before SBRT. Abbreviations: ECOG, Eastern Cooperative Oncology Group; SBRT, stereotactic body radiotherapy; CA 19-9, carbohydrate antigen 19-9.

**Table 2:** Treatment Characteristics and Dosimetric Parameters (N = 473).

Variable	Value
Radiotherapy technique	
Conventional SBRT	378 (79.9%)
MR-guided radiotherapy (MRgRT)	95 (20.1%)
Most common prescription	45 Gy in 5 fractions
Prescribed dose range	24–67.5 Gy
Number of fractions	3–6
BED10 category	<80 Gy vs. ≥80 Gy
Most common BED10	85.5 Gy
GTV-to-PTV margins	
MRgRT	Median 3–5 mm (range)
Conventional SBRT	Median 5–7 mm (range)
Respiratory motion management	Active breathing control, respiratory monitoring, abdominal compression, or equivalent institutional strategies
Daily image guidance	CBCT for conventional SBRT; real-time cine MRI for MRgRT
Online adaptive planning	Available for MR-guided treatments
Duodenal V33 <0.5 cc	
MRgRT	91.0%
Conventional SBRT	74.0%

Abbreviations: SBRT, stereotactic body radiotherapy; MRgRT, magnetic resonance-guided radiotherapy; BED10, biologically effective dose (α/β = 10 Gy); GTV, gross tumor volume; PTV, planning target volume; CBCT, cone-beam computed tomography; MRI, magnetic resonance imaging.

**Table 3:** Clinical Outcomes, Radiological Response, and Toxicity (N = 473).

Variable	Value
Median follow-up (months)	13.2
Survival outcomes	
Median overall survival (OS), months	18.4
12-month OS	62.0%
24-month OS	28.0%
Median progression-free survival (PFS), months	10.2
12-month PFS	41.0%
24-month PFS	19.0%
12-month local control (LC)	82.0%
Radiological response (RECIST 1.1)	
Complete response (CR)	21/422 (5.0%)
Partial response (PR)	117/422 (27.7%)
Stable disease (SD)	187/422 (44.3%)
Progressive disease (PD)	97/422 (23.0%)
Disease control rate (CR + PR + SD) *	325/422 (77.0%)
Biochemical response	
CA 19-9 reduction ≥50% **	60.0% of evaluable patients
Acute toxicity (n = 455 evaluable patients) ***	
Grade 0–1	401/455 (88.1%)
Grade 2	37/455 (8.1%)
Grade ≥ 3	17/455 (3.7%)
Late toxicity (n = 405 evaluable patients) ****	
Grade 0–1	375/405 (92.6%)
Grade 2	18/405 (4.4%)
Grade ≥ 3	12/405 (3.0%)
Major gastrointestinal complications (ulceration, perforation, bleeding)	None reported

Abbreviations: OS, overall survival; PFS, progression-free survival; LC, local control; CR, complete response; PR, partial response; SD, stable disease; PD, progressive disease; CA 19-9, carbohydrate antigen 19-9. * Radiological response assessment according to RECIST v1.1 was available for 422 evaluable patients. ** Biochemical response was assessed only in patients with available CA 19-9 follow-up information according to institutional practice. *** Acute toxicity data were available for 455 evaluable patients; 18 patients were not evaluable for acute toxicity assessment. **** Late toxicity data were available for 405 evaluable patients; 68 patients were not evaluable for late-toxicity assessment.

## Data Availability

The data underlying this study cannot be made publicly available due to institutional and national regulations on the handling of anonymized clinical information. Aggregated data may be provided by the corresponding author upon reasonable request and subject to approval by the participating institutions.

## Abbreviations

The following abbreviations are used in this manuscript:

SBRT	Stereotactic Body Radiotherapy
MRgRT	Magnetic Resonance-Guided Radiotherapy
BED	Biologically Effective Dose
BED10	Biologically Effective Dose (α/β = 10)
LC	Local Control
OS	Overall Survival
DFS	Disease-Free Survival
PR	Partial Response
SD	Stable Disease
PD	Progressive Disease
CTV	Clinical Target Volume
PTV	Planning Target Volume
OAR	Organ at Risk
GI	Gastrointestinal
CTCAE	Common Terminology Criteria for Adverse Events
IGRT	Image-Guided Radiotherapy
MRI	Magnetic Resonance Imaging
CT	Computed Tomography
PET	Positron Emission Tomography
CA 19-9	Carbohydrate Antigen 19-9
